# The Influence of Alcohol on Tuberculosis

**Published:** 1857-04

**Authors:** 


*The Influence of Alcohol on Tuberculosis.*

Our Address on this subject, read to the Illinois State Medical Society at its last annual meeting, has been much more extensively copied and favorably noticed by the medical press of the country than we had any reason to anticipate. But now and then an editorial *confrere* takes us all aback, and disposes alike of our facts, figures, and theories with a *dictum* worthy of *Sir Oracle.* As a specimen we copy the following from the March number of the *Charleston Medical Journal and Review:*

'Dr. N. S. Davis, the President of the Illinois Medical Society, in his Address on the "Influence of Alcohol on Tuberculosis," takes occasion again to express his very erroneous notions, on the *modus operandi* of the alcoholic stimulants.

'In answering the inquiry, what are the effects of alcohol on the human system? he has come to the following conclusions:—

"1st. The presence of alcohol in the blood produces a temporary exhilaration or excitement of the nerve structures.

"2d. It diminishes the excretion of carbonic acid from the lungs; diminishes the change of color from venous to arterial blood, and diminishes generally the organic changes in the system.

"3d. It depresses the temperature of the body, and lessens the tone of the muscular structures.

"Some of the audience may be ready to ask, if I would deny the almost universal sentiment of mankind, by claiming the broad ground, that *alcoholic liquors are never tonic, invigorating or supporting to the human system? I answer unhesitatingly, Yes!* and base my answer, not merely on the deductions of the most careful and varied experimental researches, but ask, in return, what there is in the tottering and unsteady step, the impaired digestion, the frequent functional derangements of the kidneys, exhibited, in a greater or less degree, by all habitual drinkers, which is indicative of increased physical strength, vigor, or power of endurance?"

'Here we find, not only the deliberate expression of an opinion, in direct opposition to the experience of the most learned and acute observers, but a repetition of the stereotyped error, of confounding under one name the various and diverse products of fermentation and distillation, and also of confounding the abuse with the use of these diverse agents. Dr. N. S. Davis, as Professor of the Practice of Medicine in the Rush Medical College, and Editor of the *North-Western Medical and Surgical Journal*, with six lines of titles appended to his name, must, be an influential member of the profession in the North-West, and his opinions must there obtain some weight; but we do not, even for all this, deem it necessary to undertake a refutation of the views above expressed, but leave them to suggest their own reply.'

We cannot refrain from expressing the wish that the erudite editor of the *Charleston Journal* had descended from the task of counting the 'lines of titles appended to his [our] name,' and informed us specifically which of the three propositions quoted by him are included in the catalogue of '*very erroneous notions*.' We presume it is not the first, for we are not aware that any one denies the exhilarating or stimulating influence of alcohol on the nervous system.

Is it the second? If so, he must deny the direct analytical experiments of Drs. Prout, Bouchardet, Magnus, Bocker, and some dozen other experimenters of acknowledged authority in Europe and America. But, perhaps, in his estimation, the results of direct experimental researches are not to be regarded when they chance to stand in 'opposition to the *experience* of the most learned and acute observers.' Can our Charleston friend tell us how far the 'experience of the most learned and acute observers' in Harvey's day corresponded with the results of his experiments in relation to the circulation of the blood? Or will he tell us how long it is since the '*experience* of the most learned' in the profession was all given in favor of keeping consumptive or tubercular patients carefully protected from cold air and exercise, and restricting them to sedatives, counter-irritants, and low diet? Does the history of medicine show, that what is called 'experience,' even of the 'most learned and acute,' can be safely regarded as infallible?

But it is probable that our 'very erroneous notions' are contained in the practical inferences drawn from the three fundamental propositions stated, rather than in the propositions themselves. Our chief inference was, that an agent which, while it excites the nervous system, also directly lessens the interchange of carbonic acid gas and oxygen in the lungs, diminishes the organic changes in and eliminations from the body, and indirectly diminishes the temperature, could not be either tonic or invigorating to the human system under any ordinary circumstances. Believing tuberculosis to result from a defective condition of the assimilative and nutritive functions, we did not see clearly how alcohol could act either as a preventive or curative agent in the treatment of that disease. Wishing, howewer, to test all inferences by actual facts, we canvassed the domain of medical literature on the one hand, and kept a careful record of what transpired under our own observation on the other. From the first we found many general facts of much interest, some of which we would commend to the careful consideration of our friend of the *Charleston Journal*:—

*First*, The general statistics of mortality show, that those communities and nations in which the greatest quantities of alcoholic beverages are consumed, present the highest ratio of mortality from pulmonary and tubercular diseases.

*Second*, In all the large cities where the consumption of alcoholic liquors is well known to be much greater in proportion to the population than in the country districts, the ratio of mortality from tubercular diseases is also much in excess.

*Third*, The army statistics of both England and America show, that in the armies of both countries, made up mostly of young men taken originally from the more hardy and healthy classes of society, and nine-tenths of whom, after their enlistment, have their regular rations of alcoholic drink daily, the ratio of deaths from tubercular consumption largely exceeds that of the ordinary population of the countries to which they belong.*

*Fourth*, Wherever statistical comparisons have been made concerning the absolute sickness and mortality among bodies of men of the same class, and living under the same circumstances, except that one part of them use alcoholic drinks and the other do not, the result has been uniformly in favor of the latter.†

*Finally*, We turn to a careful record of such individual cases as have passed under our own observation, and find here too no evidence of either the prophylactic or curative effects of alcoholic liquors in tuberculosis; but, on the contrary, a higher ratio of its prevalence among those who use such liquors than among those who do not.

And what does our Charleston critic offer us as an offset to all these facts, general and special? Does he adduce facts of an oppostte bearing, or tell us where such can be found? Not at all. But, instead thereof, we are flippantly told that our 'very erroneous notions' are opposed to the 'experience of the most learned observers.' Now, what is 'experience' in medicine, whether of learned or unlearned observers? Is it the mere *opinion*—the *ipse dixit* of this man or that one? Or is it legitimately the results or conclusions drawn from a careful observation and comparison of all the known facts relating to any given subject? We claim it to be the latter; and, hence, that mere *opinions* and genuine *experience* are often widely diverse from each other. For instance, Dr. Geo. B. Wood, in his recent work on *Therapeutics and Pharmacology*, says, 'It seems to be well proved, that a laborer can do a certain amount of work with less ordinary aliment, if freely supplied with beer or wine, than when water alone is allowed for drink.' This is Dr. Wood's opinion. And he tells us that *it seems to be well proved;* but he neither states the proof, nor informs us where it is to be found, and hence it can be regarded as nothing more than an expression of his belief. On the other hand, true

---

* See Dr. Drake on Diseases of the Interior Valley, &c. (*Chapter on Consumption*).

† See Statistical Tables by Mr. Waring, in the March number of this *Journal*.

'experience' in relation to this point would be the result of a certain number of actual trials, in which the food, the drink, and the labor of one set of laborers had been carefully compared with those of another set. Nothing of this kind, however, is given, either by Dr. Wood, or Dr. Happoldt of the *Charleston Journal.* The nearest approximation to it which we have seen, was contained in the statistics derived from an examination of the results of laborers in some of the large brick manufacturing establishments of England a few years since. Such examination showed, that, of several hundred laborers, a part took regularly with their meals a cup or mug of *Beer*, and the remainder took nothing containing alcohol. A record had been kept, showing the amount of each man's labor daily throughout the season. On footing up this record, it was found that the number of brick actually moulded during the season by those who abstained entirely from beer and all other alcoholic liquors, averaged, per man, several thousand more than by those who took their regular rations of beer. The former had also enjoyed a greater immunity from sickness than the latter. Similar inquiries in relation to men employed in almost every department of human industry, have developed the same relative results.

Now, if alcohol is truly tonic or invigorating to the human system; if a laborer who drinks beer can do a given amount of work with less food than one who drinks only water, how are the above facts, derived from careful and extensive statistical researches, to be accounted for? Accounted for! did we say? Why, very easily indeed. Our Charleston friend does it in a single sentence, by simply alledging that we have committed the '*stereotyped error* of confounding under *one name* the various products of fermentation and distillation, and also confounding the *abuse* with the *use* of these various agents.' Inasmuch as my Address to the State Medical Society was on the Effects of *Alcohol* as a Prophylactic and Therapeutic Agent in the Prevention and Treatment of Tuberculosis, it is not easy to appreciate the bearing of the first charge in the quotation just given. Does the editor of the *Charleston Journal* mean to convey the idea that alcohol produced by fermentation is different from alcohol derived from distillation? If so, we plead guilty to the charge of having 'confounded' them with each other; for, in our blissful ignorance, we had supposed that alcohol was properly and exclusively the product of vinous fermentation, while distillation only served to separate it from some of the accidental ingredients with which the fermentation had left it mixed. It is quite possible, however, that our critic simply meant to convey the idea that some of these accidental ingredients modified the effects of the alcohol, which is doubtless true to a very limited extent. But we ask in all candor, if it is not the *alcohol* in all the various liquors which gives them their essential qualities, and for which they are prescribed and drank?

Does Dr. Happoldt, or any other physician, prescribe or use beer, merely for the *hops* it contains; or wine, for the vegetable acids mingled with it; or gin, for its juniper; or corn whisky, for its fusil oil? If he does, we must enter a decided protest against the justice and propriety of the practice. We take it to be a settled axiom, that a physician is bound to use, as far as possible, medicinal agents of known purity and uniformity. But will any sane man pretend that we cannot get hops, and vegetable acids, and juniper, and fecula, and even fusil oil, directly from reputable druggists in a far more pure and reliable state than from the 'varying' and generally grossly adulterated products of the brewery and distillery?

We must hasten, however, to the second and last charge of our critic, viz.:—That our investigations are rendered 'erroneous' and worthless from our having 'confounded the *abuse* with the *use*' of alcoholic drinks.

This reply, if not '*stereotyped*,' has certainly been a *standing* one with the advocates of alcoholic drinks for the last half century. It matters not what view we take, or what course of investigation we pursue, the reply ever comes back the same. If the moralist points to the almost universal wreck of mind and body produced by the *use* of drinks containing alcohol: the reply is, 'You confound the *abuse* with the *use*.' If the political economist points to the lost fortunes, the squalid poverty, the social degradation, and the political corruption produced by these agents: the same echo comes back, 'Oh, Sir, you *confound* the *abuse* with the *use* of these agents.'

Drs. Prout, Bouchardet, Magnus, Bocker, and a host of others, devise and execute varied experiments on the secretions, excretions, and temperature of the human system, while influenced by alcoholic liquors: but to the legitimate inferences drawn from all their work, the same charge comes to the ear with scarcely a variation in the quality of sound. Dr. Bocker, in his experiments, used the alcohol in doses of about one *fluid drachm;* in ours, we used wine in quantities varying from two to eight ounces, and brandy in doses of from one to four ounces. In the cases of phthisis we detailed in our Address, some had used beer in quantities varying from one to four or five glasses per day; some had used from one to three moderate drinks of distilled spirits per day for years, but never sufficient to intoxicate; while others had used all kinds and qualities indiscriminately, and to frequent intoxication: yet, in reply to all these, comes still that same ubiquitous, stereotyped, threadbare charge of 'confounding abuse with use.' With all due deference, we protest against the continuance of this course as unworthy of any man who claims to cultivate medical or any other science. If our friend at Charleston, or any one else, will present reliable statistics, or the results of well executed experiments, which fairly contradict or correct those already on record, we will most cheerfully accord to them their true value.

